# Three contextual dimensions of information on social media: lessons learned from the COVID-19 infodemic

**DOI:** 10.1007/s10676-020-09550-2

**Published:** 2020-08-26

**Authors:** Lavinia Marin

**Affiliations:** Ethics and Philosophy of Technology Section, TU Delft, Delft, The Netherlands

**Keywords:** Disinformation, Infodemic, Covid-19, Designed interaction, Online emotions, Context sensitive design

## Abstract

The COVID-19 pandemic has been accompanied on social media by an explosion of information disorders such as inaccurate, misleading and irrelevant information. Countermeasures adopted thus far to curb these informational disorders have had limited success because these did not account for the diversity of informational contexts on social media, focusing instead almost exclusively on curating the factual content of user’s posts. However, content-focused measures do not address the primary causes of the infodemic itself, namely the user’s need to post content as a way of making sense of the situation and for gathering reactions of consensus from friends. This paper describes three types of informational context—weak epistemic, strong normative and strong emotional—which have not yet been taken into account by current measures to curb down the informational disorders. I show how these contexts are related to the infodemic and I propose measures for dealing with them for future global crisis situations.

## Informational disorders on social media relating to COVID-19

From the beginning, the pandemic was reflected in the online realm of social media by a flood of redundant information (the so-called infodemic) out of which a significant percentage was made up of misinformation and disinformation (MDI). MDI is used here as one umbrella term for two distinct phenomena: misinformation, which is usually defined as false information shared without knowledge that it is false, while disinformation is fabricated information distributed with the clear intention to mislead (Fallis 2014, p.136). However, on social media, these distinctions are hard to maintain sharply, since we cannot always know who created a piece of misleading information and with what purpose. The infodemic is understood as “an overabundance of information—some accurate and some not—that makes it hard for people to find trustworthy sources and reliable guidance when they need it” (World Health Organization 2020). This concept is similar with that of information pollution which was defined as “irrelevant, redundant, unsolicited and low-value information” (Wardle and Derakhshan 2018, p.10) but applied to a crisis situation when an infodemic can become dangerous (Tangcharoensathien et al. 2020, p. 2).

Already since February 2020, the World Health Organization had singled the infodemic as an emerging problem in the context of the COVID-19 pandemic. One of the most problematic aspects of an infodemic is that it creates information overload which leads to information fatigue for online users: the user’s capacity for paying attention to information is limited and tends to exhaust quickly. Studies in the psychology of social media have shown that, under conditions of informational overload, users will revert to using mental shortcuts or heuristics for assessing new information. By employing cognitive heuristics, social media users tend to rely on their friends and their endorsements in selecting what information to trust or engage with (Koroleva et al. 2010, p. 5). Taking these mental shortcuts occurs “in conditions of low motivation and limited ability to process the incoming information” (Koroleva et al. 2010, p. 5) which is arguably the case when dealing with information overload. Trying to save their mental energy, users will tend to delegate their critical engagement to their social network, trusting their peers, or to become disengaged from so many news and revert to apathy. Both strategies are dangerous because social media users are also citizens who are instrumental in the efforts to curb down the pandemic. If citizens do not correctly understand what they have to do in a pandemic situation, then governmental measures will be ineffective.

Online information about the COVID-19 pandemic posted on social media displayed two seemingly distinct problems: the rapid propagation of misinformation and disinformation (MDI) and the so-called infodemic. Both problems concerned how information travels in an online social networking medium, but only one of them was tackled with some degree of efficiency. Social media platforms rapidly stepped up their pre-existing measures of dealing with MDI and targeted specifically the COVID-19 related misleading information, whereas the infodemic remained untouched (Howard 2020). The infodemic was seen as a side-effect of the intensification of user interactions on social media, some informational noise that accompanied the humming of online communications.

In this article, I will argue that both informational problems are related with and symptomatic of a deeper problem embedded in social media: the contextual design of the user’s interactions with information. While we certainly need to pay attention to the quality of informational content (Floridi and Illari 2014) distributed on social media, similar attention should be paid to the quality of the user’s interaction with the informational context. Measures focusing solely on the factual content of information distributed online risk ignoring a significant aspect of social media, its particular context of engaging with information. The paper is structured as follows: first, I briefly review the measures taken by social media platforms to deal with the COVID-19 related MDI, classifying them in view of the content or context focus. Secondly, I describe three dimensions of the informational context on social media which made MDI particularly difficult to deal with during the pandemic and, by extension, aggravated the infodemic. Finally, drawing from the contextual approach to information, I propose some measures to tackle informational disorders online for future similar global crisis situations.

## Counter-measures against mis-/dis-information related to COVID-19 on social media

MDI related to COVID-19 was tackled visibly by most mainstream social media platforms. This was possible because there were already some methods in place for dealing with MDI. Starting with the 2016 elections and the Cambridge Analytica scandal, social media platforms began paying attention to the MDI shared by their users. In recent years, social media platforms have been testing methods of content curating by using external fact-checking organisations, flagging the misinforming content, and sometimes removing it (Howard 2020). In the wake of the COVID-19 pandemic, these efforts for fact-checking were accelerated to an impressive extent: “the number of English-language fact-checks rose more than 900% from January to March” (Brennen et al. 2020). This concentration of effort from disparate organisations was motivated by the emergency of the situation but also by the topic of COVID-19 which rendered itself easier to fact-check: it was more or less clear what was false content^1^ and what not—as opposed to previous cases of political MDI (Brennen et al. 2020).

Table 1 below summarises the main measures taken by social media platforms for dealing with MDI during the pandemic.^2^

**Table 1:** Main types of measures against MDI on social media during the pandemic

No.	Counter-measures	Approach to MDI	Type of approach	Platforms implementing it
1	Flagging misinformation by regular users—double-checked by editors later	Decreases the quantity of MDI	Content-focused	Facebook, Twitter
2	Fact-checking by certified third parties	Decreases the quantity of MDI, hence the user’s interaction with it	Content-focused	Facebook, Twitter
3	Less visibility for repeated offenders (users who distribute MDI) in the news feed	Decrease in visibility of MDI, hence the user’s interaction with it	Content-focused + Context-focused	Facebook, YouTube, Reddit, Twitter
4	Deleting accounts of MDI creators and bots	Decreases the quantity of MDI	Content-focused	Facebook, YouTube, Twitter
5	Adding a visible link to official information on COVID-19 from WHO	Increases visibility of accurate information, presumably educates the users	Content-focused + Context-focused	Facebook, YouTube, Twitter
6	Creating a dedicated page for local news and official information on COVID-19, linking to it visibly	Increases visibility of accurate information, presumably educates the users	Content-focused + Context-focused	Facebook, Twitter
7	Displaying “related” items below posts about COVID-19, most of which are leading to fact-checked information	Increases diversity of the information to which the user is exposed, presumably educates the users	Context-focused + Content-focused	Facebook, YouTube

Most approaches listed in the previous table were primarily content-focused as they were targeting the factual or descriptive content of the information, by checking it against existing evidence and reducing its visibility. However, the context of MDI is just as important as its content (Wardle and Derakhshan 2018). Contextual MDI may appear when placing genuine information in a fabricated setting, for example, a private statement cited as if it were a public one, a personal opinion as to represent the views of an organisation, mixing facts with irrelevant comments, or changing the date or place where a photo was taken. A large extent of COVID-19 related MDI shared on social media was contextual or, as some have put it, reconfigured: “most (59%) of the misinformation in our sample involves various forms of reconfiguration, where existing and often true information is spun, twisted, recontextualised, or reworked. Less misinformation (38%) was completely fabricated” (Brennen et al. 2020). Even those measures which combined content with context awareness (such as numbers 3,5,6 and 7 in the Table 1), the content was the primary focus. These measures relied on someone checking first what information was good enough and then proposing some contextual measures.

Content-focused approaches are not misguided, but tend to give most of the agency to the social media platform, while the users are left with a passive role, to click and react to what they are shown. Meanwhile, when a measure is both content and context focused, the user’s agency starts to play a significant role. No matter how many alternative sources of information one is exposed to (as it is the case with measures 5,6,7 in the Table 1), the user has still the final choice to engage with them or not. By contrast, a context-focused approach would presuppose that the user has the freedom to use the social media platform in a way that stimulates and recognises other kinds of engagement with information, regardless whether the information at hand is genuine or MDI. A context-focused approach creates informational environments which accommodate the possibility that the users may engage differently with and interpret differently the same information. This approach would aim to stimulate the users in becoming more sensitive to the modes in which the information is presented to them, educating them in the long run.

This notion of context-sensitivity was inspired by similar constructs encountered in Human–Computer Interaction studies such as “contextual design” (Wixon et al. 1990) and “context-aware computing” (Schilit et al. 1994–1994). Context sensitivity in design starts from the idea that the user’s perspective is not that predictable, and that there is not one single context of use, accepting that users make sense of an interface in various ways, hence proposing that the designer accommodates for multiple meanings and complex interactions right from the start. In the long run, a contextual approach can educate the users without trying to change their beliefs or attitudes about the informational content as such. The contextual information approach is inspired by Value Sensitive Design (Friedman 1996; van den Hoven 2007) which is complemented by paying attention to three particular dimensions of informational context on social media, described in the next section.

## Three dimensions of the contextual information on social media

### The strong emotional context

Before the pandemic, it was already shown that MDI propagates on social media platforms by playing on the emotional reactions of the online audience (Zollo et al. 2015; Khaldarova and Pantti 2016), aiming to deliberately stir powerful emotions in their readers. Some researchers called this feature of MDI a form of “empathic optimisation” (Bakir and McStay 2018, p. 155). Emotional manipulation in news items (especially click-bait) is an efficient way of capturing user’s attention since emotion-stirring news are usually more interacted with than the neutral ones (Bakir and McStay 2018, p. 155). It may seem that only MDI is emotionally loaded, whereas genuine news sound more sober and neutral. But this would be a misleading view of how information travels on social media platforms. Emotional reactions do not belong to misleading information alone, rather these are a normal side-effect of the emotional infrastructure already embedded in most social media platforms.

**Fig. 1:**
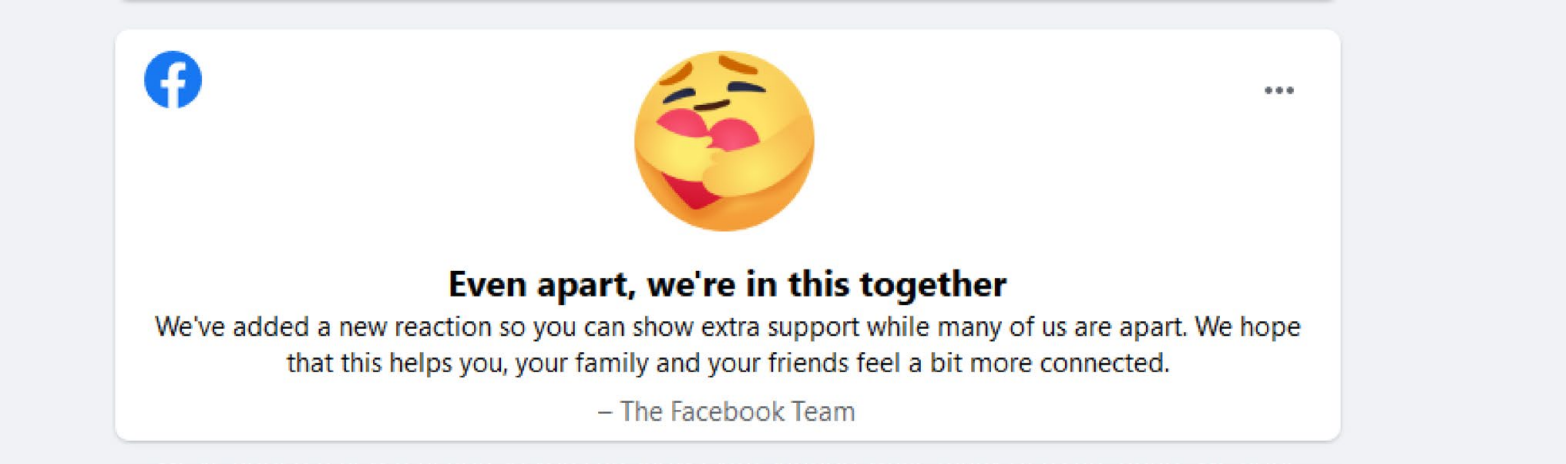
Facebook adds a new emotional reaction in the wake of Covid-19

Users of social media platforms are allowed a palette of actions and reactions: some are seemingly neutral (commenting, sharing and posting) while others have a clear emotional valence: liking and using other emoticons to endorse or dislike a post. These emotionally charged reactions are easier to perform than the neutral ones: it takes a split second to click like on a post, but some more time to comment on it or even share it. Most of these emotional reactions have dedicated buttons which can be clicked mindlessly, yielding the interaction seamless. In the wake of the Covid-19 pandemic, Facebook even added a new emoticon expressing a reaction of solidarity (see Fig. 1). The assumption was that now, more than ever, users needed to express emotions online with a richer palette. However the simplistic way of expressing such emotions did not change, it was part of the interaction design from the beginning.

The emotional infrastructure of social media was not something requested by users but designed from the start. Major social media platforms are oriented towards maximising the user’s engagement, i.e. how much time one spends on a specific platform and how much attention is consumed (Whiting and Williams 2013). These kinds of interactions actively promote the user’s “attention bulimia” i.e. a behaviour oriented towards “maximizing the number of likes” (Del Vicario et al. 2016, p. 1) and presumably other positive reactions. Most buttons for emotional reactions are of positive emotions (like, love, hug, laugh) while in recent years Facebook added some more nuanced emotions such as angry, sad or cry. But the overwhelming effect of these emotional reactions is to make other users feel liked by their social network hence, to make the platform a place where one wants to keep returning to for emotional gratification.

For many users confined to their homes by the pandemic, social media platforms became a window to the world, as the television set used to be in older days and the easiest way of relating to others. In such times of distress and uncertainty, users posted more frequently than usual (Cinelli et al. 2020) but some of the information posted was not meant to inform others, but rather to express one’s concerns and emotions related to the pandemic. Posts were also meant to get reactions from one’s friends in an attempt to confirm that the others were also feeling the same way as one does. Posting about the pandemic became a strategic way of gauging other’s emotions on the crisis situation and gathering some feeling of consensus from one’s social network. The consensus sought on social media was of an emotional nature which may be at odds with an epistemic consensus about the nature of the facts at hand.

### The weak epistemic context

During the pandemic, several epistemologists and philosophers of science stepped up and tried to educate the general public on what sources to trust as experts and how to discern facts from fiction about the pandemic—in podcasts, opinion pieces and on their social media accounts (Weinberg 2020). While this effort is laudable, it needs to be complemented with another approach, taking into account the wider epistemic context in which information travels on social media. This is a particularly weak epistemic context in which information is not always shared to inform. Social media platforms are not places where one usually goes to be informed. At least in regular, day to day situations, users turn to social media platforms to relate, to communicate and to be entertained (Fuchs 2014). The weak epistemic context of social media is ruled by serendipity (Reviglio 2017), meaning that many users get to be informed by accident.

In a crisis situation, users tend to change how they use the platform and shifting towards the communication of vital information such as imminent risks or their location and also seeking to be informed by latest developments from people from the local site of the disaster. The entertainment function tends to become secondary in emergencies (Zeng et al. 2016). In the 2020 pandemic situation, the difference was that the crisis was global and that the duration was rather long. This time, the uncertainty that accompanies a crisis situation was extended over months. As epistemic agents, online users tried to make sense of what was going on with them, what they could expect and to assess the personal risks, over a longer period.

**Fig. 2:**
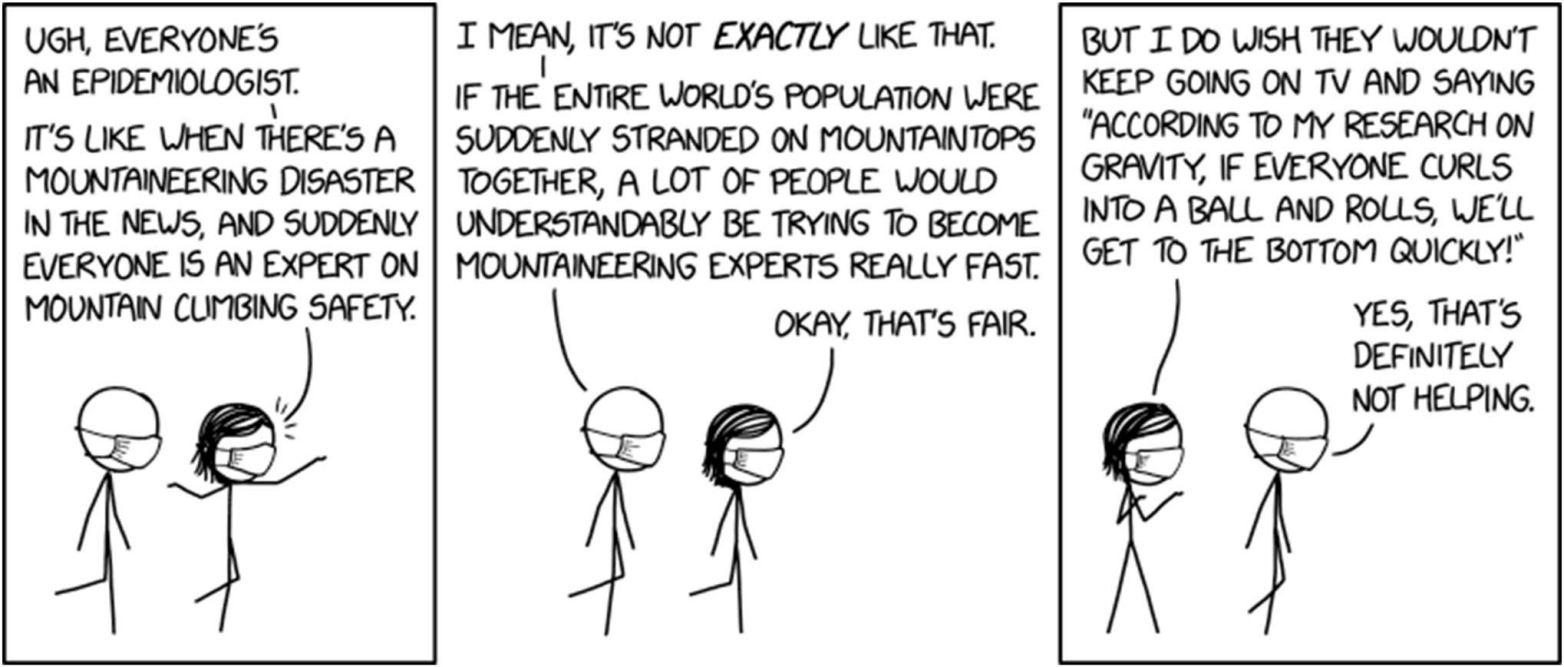
Everyone is an expert. Image source: https://xkcd.com/2300/

The pandemic was an extended crisis situation compounded with social alienation on top. This made users feel lost and overwhelmed by problems one could not understand. Hence the desire—legitimate to a point—for everyone to be an expert so that they could at least understand what was happening to them. People did not want to be experts in epidemiology, quarantine measures, and home remedies for viruses because of a sudden surge of intellectual curiosity. They needed a way of coping that was also understandable to them. Meanwhile, the official discourse of “trust the experts” and “please don’t share information you do not understand” incapacitated them as epistemic agents. Requiring users to do nothing and just comply went against the general desire to do something, as a way to take control. Given the increase in posts on the pandemic by regular users, it may seem that many have tried to become experts overnight in epidemiology, viruses and vaccines. The comic below (Fig. 2) illustrates the frequent situation emerging during the pandemic of members of the lay public hijacking the role of the expert.

A discussion on conditions of trust and expertise makes sense in a regular epistemic context when agents try to acquire knowledge about a domain they know nothing about, having to choose which experts to trust (Goldman and O’Connor 2019). However, acquiring knowledge was probably not the main goal of social media users who started posting scientific information which they did not understand. Rather, many users tried to build some understanding of the situation, to make sense of the events. In these cases, shared understanding in a circle or network of friends seemed to be more important for users than gaining access to expertise. The scientific information was posted by lay users to back one’s personal opinions, to urge for a certain course of action, or to gather consensus. Given these weak epistemic uses of information targeted at emotional fulfilment and networking purposes, the regular content-focused measures would probably be less effective than predicted on such users.

### The strong normative context

Related to the previous point and stemming from it, most factual information shared around on social media had some normative implications which often shadowed any knowledge claim. Descriptive information was used for prescriptive or evaluative aims. Scientific expertise was co-opted strategically to enforce one’s own pre-existing evaluative opinions. Typical MDI claims are not merely descriptive claims of a state of affairs in the world, but often embedded in a normative context be those prescriptive or evaluative claims, both types are meant to change attitudes of the online users. MDI was shared because it prescribed actions or led to evaluations of the state of affairs which users already agreed with. Hence debunking the facts would have solved only half of the puzzle, since the user’s motivation to believe these normative claims would have not been dealt with.

One example of the strong normative context for MDI, also involving a clear “politicisation” of MDI during the pandemic (Howard 2020), concerns one of the most popular types of claims analysed by EU vs Disinfo (2020) in which the EU was depicted as powerless and scattered in dealing with the pandemic. This claim was traced back to Russian-backed agencies which aimed to make users believe that, ultimately, Russia was stronger than the EU (Howard 2020). Such claims can be debunked by showing that there were coordinated measures taken by the EU, however, the implicit claim that other states dealt better with the pandemic than the EU is hard to debunk since it is not explicitly stated. This is just one type of difficulty with MDI which cannot be tackled with a content-focused approach: implicit evaluative claims in which one term of the comparison is not named.

Some evaluative claims can be checked (if these involve relational predicates which are measurable such as “better than” or “more efficient than”) however others, incidentally the politicised evaluative claims, are harder to assess. In Russian-backed claims against the EU, the name of “Russia” is not mentioned anywhere in the text of the “news”, since the aim is to erode the trust in EU from its citizens. If these citizens happen to be in Eastern Europe, this erosion of trust could lead to an anti-EU generalised feeling, and ultimately bottom-up pressures to exit the EU. These kinds of campaigns cannot be easily fact-checked since the effect is achieved by playing the long game. What looks like news about the pandemic is a dog-whistle about something else.

The strong normative context is visible also when using scientific expertise co-opted to back up prescriptive claims otherwise untenable. One example is an unpublished paper by Blocken et al. titled “Towards aerodynamically equivalent COVID19 1.5 m social distancing for walking and running” (2020) in which an animated image showed a simulation of how joggers coughing will spread particles of droplets when running at a distance of 1.5 m from each other. The paper became viral on social media despite not being peer-reviewed nor published on a pre-print website. The visual animation showing the spread of droplets was understandable by every lay member of the public, without needing to have specialised knowledge of actual aerodynamics, and presumably made the paper so popular among non-scientists who used it to make prescriptive claims by non-scientists. While the authors hypothesised that it might be unsafe to run close to another—and that even 1.5 m distance might not be enough for jogging—the social media audience took this as a reason to shame the runners in their neighbourhoods (Koebler 2020). Meanwhile, the first author of the study posted a document on his website answering certain questions about the study^3^ and refused to draw any epidemiological conclusions, urging for other’s expertise. But social media users did not shy from becoming experts and drawing the conclusions themselves, as the information in the Blocken et al. paper was just ammunition in a larger informational battle about what others should do.

Even if the scientific claims of regular users are checked, their aim remains to prescribe actions for others and to evaluate the world in a way that will be endorsed by one’s community of friends. For these purposes, other pieces of news will be co-opted if the first ones were flagged as hoaxes. Content-based approaches are then ineffective against this strong desire of social media users to emit evaluative or prescriptive claims about the world and strategically use science-looking sources to back these up. One should address the very desire of regular users to evaluate the world from the little soap-boxes that social media affords.

## Avenues for future development: contextual approaches to informational disorders online in times of crises

The three dimensions of the informational context on social media previously mentioned (strong emotional, strong normative and weak epistemic) have been analytically distinguished but they function simultaneously to promote certain user behaviours which one could call irresponsible information sharing on social media. The weak epistemic context re-enforces the strong normative claims which are coupled with emotional reactions leading users to become strongly attached to their claims and indifferent to their debunking. While the three dimensions of the informational context work together to produce a perfect storm of low-quality and redundant information—an infodemic—one could still try to design modes of interaction to deal with each of them.

Future measures dealing with MDI and infodemic in a global pandemic need to also tackle the high emotional load of most MDI items. A practical way of flagging this could be devised by modelling on Facebook’s “hoax alert” system which warns users that a certain post they are reading has been fact-checked and is probably a hoax. A similar system could be designing an “emotional alert” flag which appears below certain news-looking items which have an unusually high amount of emotional triggering words. This kind of alert would show to users when certain news-like items lead them to feel something quite specific. The readers would be still free to engage with such items, but at least they would be warned about possible emotional manipulation. Such alert would also flag click-bait and irrelevant news which are not false in themselves, but which do contribute to the infodemic because of the high virality and potential for polarisation.

The weak epistemic context needs to be tackled together with the high normative one. When users engage with news-items relating to the pandemic (or any other crisis situation), they can be shown alternative news and pages or users to follow which are experts in the field. This has been already implemented, but with little success as it needs to be complemented by more context-aware measures. A new measure could be to try to increase the users’ critical engagement with a certain class of news items. This could happen by posting a small survey under the tricky news items asking the users to answer the following questions: “What does this news lead me to believe?”, “What does this post ask me to feel?”, “Please rate how strongly you agreed with these claims before reading this post”. And, finally, after answering this mini-survey, the user could be shown a disclaimer stating, “Now that you’ve read posts you agreed with, how about trying something different?”—and the alternative sources with diverse information from experts could be displayed, but after the user has been primed to be more critical and diversify one’s information sources. To incentivise users to fill in these surveys, they could receive certain bonus points on the social media platform and, once they accumulate a certain number of points, they could get a badge next to their name of “critical media user” or “critical thinker” which would ensure visibility of their informational skills.

These sample measures I have proposed—and presumably other context-sensitive measures—are fit to be implemented only in crisis situations and only targeting the user’s posts about the current crisis situation. It is, of course, possible to integrate these measures in the day to day users’ interaction. However, social media fulfils certain deep emotional needs of users such as expressing strong normative opinions and seeking emotional consensus. If these contexts are strongly discouraged, the users may find other platforms to do so and they may abandon the too critical platforms. Meanwhile, in a crisis situation, these dimensions could be targeted specifically and confined to users that share MDI and redundant information about the crisis at hand.

This paper outlined three types of informational context which have not yet been taken into account by the current measures to curb down disinformation and the amount of irrelevant information on social media platforms. MDI countermeasures taken thus far have a limited effect because these did not account for the diversity of information contexts on social media. These measures do not address the primary causes of the infodemic itself, namely the user’s need to post content as a way of making sense of the situation and of expressing one’s opinion as a way of gathering consensus reactions. In light of these considerations, we need a value-sensitive design re-assessment of how users interact on social media among themselves as well as with the information found online in crisis situations. Designing for thoughtful, critical and meaningful user interaction should become an explicit aim for the future of social media platforms in times of pandemics and other global emergencies.

## Footnotes

1 For a list of categories of false claims about Covid-19, see the dynamic graph found at https://euvsdisinfo.eu/throwing-coronavirus-disinfo-at-the-wall-to-see-what-sticks/.

2 Data sources used for this table: Silverman (April 2019), Naeem and Bhatti (2020), Cinelli et al. (2020), Tangcharoensathien et al. (2020), EU vs Disinfo. (2020) and Brennen et al. (2020).

3 https://www.urbanphysics.net/Questions_and_Answers.pdf.
